# A Comparison of Ice Cold Water Pretreatment and ****α****-Bromonaphthalene Cytogenetic Method for Identification of *Papaver* Species

**DOI:** 10.1155/2013/608650

**Published:** 2013-09-11

**Authors:** Amir Rezaei Osalou, Sheida Daneshvar Rouyandezagh, Behrouz Alizadeh, Celal Er, Cafer Sirri Sevimay

**Affiliations:** ^1^Department of Field Crops, Faculty of Agriculture, Ankara University, 06110 Diskapi, Turkey; ^2^Department of Agricultural Biotechnology, Faculty of Agriculture, Namik Kemal University, 59030 Tekirdag, Turkey

## Abstract

The plants belonging to many species in genus Papaver are very similar and have very small chromosomes that make identification very difficult. The study aimed to compare the effects of **α**-bromonaphtalene and ice cold water pretreatment to identify chromosomes of Papaver species collected from different areas of Iranian West Azerbaijan and Turkish Van, Agri and, Hakkari provinces. The seeds were germinated in Jacobson trays at 24°C under continuous light. Thereafter, roots from 1.5 cm long plantlets were pretreated with **α** bromonaphtalene for 15, 30, and 45 min or at 0°C in ice cold water for 24 h before fixing, hydrolyzation, and feulgen staining. The ice cold water pretreatment was more appropriate and easy to determine chromosomes. Seeds from seven samples did not germinate. Sixty samples out of the rest of 62 samples were identified as *P. pseudo orientale*, one sample was identified as *P. bracteatum*, and another as *P. orientale*. This is the first study that used ice cold water to determine the chromosomes in papaver species. It is hoped that it will also facilitate to determine chromosome number and identify other papver species.

## 1. Introduction

*Papaver pseudo-orientale* (2*n* = 6*x* = 42), *P. bracteatum* (2*n* = 2*x* = 14), and *P. orientale* (2*n* = 4*x* = 28) are three important species belonging to the section Oxytona, which resemble very closely. These species were first brought to Europe in the early eighteenth century by Tournefort and were introduced as “oriental poppy” [1].

*P. pseudo-orientale* grows at an altitude of 1600–2200 m at moist places in Iran and Turkey [1], which is used as an ornamental plant because of its beautiful and attractive flowers. *P. pseudo-orientale* displays erect flower buds with orange-red petals with or without basal marks. It is rich in isothebaine [2], macranthaline, orientalidine, and salutaridine, which makes it an important medicinal plant [3] of pharmaceutical importance. Concentration of these alkaloids varies in capsules, leaves, stems, and roots. The alkaloid spectrum and content also differ during plant growth and development. Thebaine is the most important alkaloid obtained from this group of plants.

Identification of the plant species is difficult, which is generally done after consultating taxonomic keys to be sure that specimen is identified correctly. However, phenotypic appearance of the three species *P. pseudo-orientale* (2*n* = 6*x* = 42), *P. bracteatum* (2*n* = 2*x* = 14) and *P. orientale* (2*n* = 4*x* = 28) is very similar and there is every likelihood that plants are identified erroneously. Therefore, another important way to determine the plant species is to determine the chromosome numbers, which are a concrete feature to remove any ambiguity among the three species of section Oxytona [1, 4]. The ploidy level provides clear delimitation of species in this section. According to a systematic study by Goldblatt [1], the hexaploid *P. pseudo-orientale* (2*n* = 42) is regarded as transient form between (or an allopolyploid hybrid of) the diploid *P. bracteatum* (2*n* = 14) and the tetraploid *P. orientale* (2*n* = 28).

Cytogenetic analyses reveal formation of multivalents at diakinesis, in the polyploid species like *P. pseudo-orientale* and in its hybrids with diploid species. This finding demonstrates the autoploid nature of *P. pseudo-orientale* and proves that *P. bracteatum* is as its ancestor. Similarly, the similarities found in isozyme variation and in chloroplast DNA restriction patterns between and within the three species of section Oxytona also strongly indicate the autoploid nature of this section [5, 6].

Producing good somatic metaphase spreads is hindered by combined difficulties of obtaining large number of dividing cells and of spreading and staining the chromosomes well. The thickness and length of chromosomes are other important factors that affect visibility of chromosomes during metaphase. It is also important to catch the best view at appropriate stage before fixation of chromosomes. Over the years, many methods have been developed for preparing somatic chromosomes, and they are generally made up of four stages: first, the seed is germinated and the actively growing root tip is collected; second, a pretreatment using ice cold water for a definite period of time or 8-Hydroxyquinoline, Colchicine, Paradichlorobenzene, or *α*-monobromonaphthalene for a definite period of time prior to fixation to inhibit spindle formation, preventing the congression of chromosomes to the metaphase plate; third is fixation in any of a large range of solutions; fourth, preparation of the root tips by hydrolysis, followed by staining.

Most studies pertaining to chromosome counts in *P. pseudo-orientale* make use of *α*-monobromonaphthalene as pretreatment. This study aimed to compare the effects of *α*-monobromonaphthalene and 0°C water pretreatment for 24 hours on chromosome visibility of Papaver species, which is an important method for observing cereal chromosomes, where this pretreatment is preferred over other methods when chromosomes of a large number of plants are to be studied.

## 2. Materials and Methods

### 2.1. Plant Material

The study included 69 different populations of Papaver section Oxytona collected from Van, Hakkari and Agri provinces of Eastern Anatolian Turkey and two samples of Iran origin, collected from West Azerbaijan Province of Iran. The seeds were germinated between moist sandwiched filter papers on Jacobson trays at 24°C under continuous light. Thereafter, when the plants increased to lengths of 1, 1.5, 2, and 3 cm, they were collected for preparation of preparates in the laboratory. At least 5 preparates were made from each sample unless specified.

### 2.2. *α*-Monobromonaphthalene Method

The roots of selected plants of sample number 414 were pretreated with *α*-monobromonaphthalene for 15, 30, and 45 min at room temperature followed by rinsing of pretreated roots with distilled water for 4-5 min.

### 2.3. Fixation

Thereafter, in order to fix or stop the chromosomes at the desired stage of cell division, the roots were treated with Carnoy's solution consisting of 1 part glacial acetic acid and 3 parts ethanol (95 to 100%). This fixative was prepared fresh each time. The material was kept in the fixative for 30 minutes at room temperature followed by rinsing with bidistilled water.

### 2.4. Hydrolysis

Then roots were hydrolysed for 8 minutes in 1 N HCl at 60°C followed by washing with bidistilled water.

### 2.5. Staining

Hydrolyzed specimens were stained with Feulgen for 30 minutes under darkness at room temperature followed by washing with bi-distilled water before making preparates.

### 2.6. Making Preparates

The roots were quickly transferred into 45% acetic acid dropped on a microscopic slide previously. Then 2-3 mm-long meristematic (well stained) sides of roots tips were cut discarding remaining portions of the roots. The well-stained root tips were carefully sliced using a sharp blade (Gillette) dropping 45% acetic acid little by little to avoid the escaping of sliced tissues from the blade and preventing drying of the acetic acid. These tissues were mixed up to get a homogenous liquid using a needle and closed slowly beginning from one side to another side by a coverslip. Thereafter, the surplus liquid was absorbed using blotting paper. To avoid the slipping of coverslip over the slide, it was kept firmly held using thumb and was carefully knocked using bottom of a pencil to flatten the cells, avoiding chromosome diffusion and facilitating easy observation of chromosomes without overlapping. Air bubbles created between glass slide and coverslip were eliminated by dropping 45% acetic acid at the edge of each coverslip. The extra-acetic acid was absorbed using blotting paper. The preparates were observed using a Nikon microscope to count and distinguish chromosomes.

### 2.7. Pretreatment Using Melting Ice (Melting Ice Method)

Germination and selection of the plants was done as suggested previously. However, the roots were pretreated by placing the selected plants at 0°C in the melting ice, inside a refrigerator at 4°C without cutting them for 24 hours.

### 2.8. Fixation

Thereafter, the roots of the plants were cut and treated with Carnoy's solution 1 consisting of ethanol-acetic acid (3 : 1) for 30 min at room temperature followed by washing with distilled water.

It was followed by hydrolysis for 8 min in 1 N HCl, at 60°C, staining with Feulgen for 30 min at room temperature, washing with bidistilled water, and preparation of slides, as described above.

## 3. Results and Discussion

### 3.1. Using *α*-Monobromonaphthalene Method for Chromosome Determination

**Figure 1:**
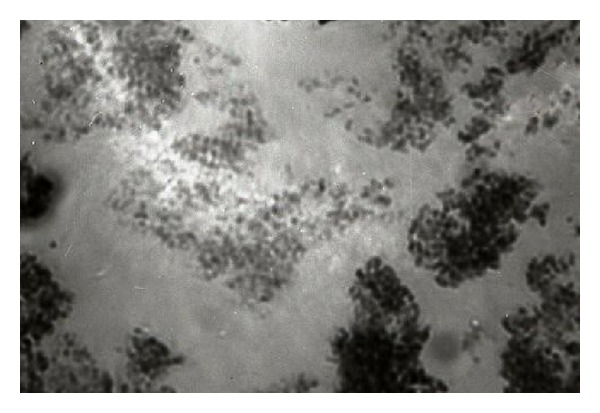
2*n* = 6*x* = 42 chromosomes belonging to sample 504 K of *P. pseudo orientale* collected from Dibekli koprusu, Van Turkey pretreated with *α*-monobromonaphthalene for 45 minutes.

A good experimental procedure has to show clear and sharp chromosomes that were not possible when *α*-monobromonaphthalene method was used. The analysis of root tip slides prepared from 1, 1.5, 2, and 3 cm long plants of *P. pseudo-orientale* using sample number 504 K collected from Yuksekova Dibekli koprusu, Turkey, showed that the species exhibited lower and blurred expressions (Figure 1). The chromosomes were difficult to count, unrecognizable, and dim. Moreover, the treatment resulted in more diffusion of chromosomes. Using *α*-monobromonaphthalene method for chromosome determination was not helpful. Martens and Reisch [7] observed that the influence of the time of sampling in the activity of cell mitosis is very important. It is thought using *α*-monobromonaphthalene method for chromosome analysis may have negatively affected the roots tips during pretreatment with monobromonaphthalene causing some internal rearrangements in the root tips that caused difficulty in observing and counting the chromosomes at the time of mitotic division of root cells. It also decreased the quality of the studied chromosome. It seemed as if *α*-bromonaphthalene method was incompetent and encountered problems in chromosome counting.

### 3.2. Using Ice Cold Water Method for Chromosome Determination

**Figure 2:**
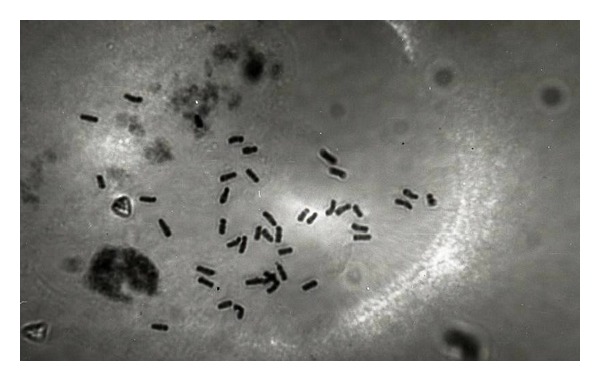
(10 × 40), 2*n* = 6*x* = 42 chromosomes belonging to sample 419 K of *P. pseudo orientale* collected from Dogubayezit-Igdir Road and Agri province, Turkey, pretreated with 0°C melting ice for 24 hours.

Optimization of method for chromosome counts was made using sample number 419 K collected from Dogubayezit-Igdir Road location, and using ice cold water method for chromosome determination was very helpful. The preprates were initially pretreated in ice cold water. The chromosomes were shorter and thicker. The results also showed better staining and separation of chromosomes, so that they were visible and countable. This elevated the opportunity of observing and counting the chromosomes easily. Irrespective of seedling length, the method could easily help in helping the chromosome counts. The chromosomes were recognizable and clear and had high expression. Moreover, they were not difficult to count under the microscope, yet the initial experiments showed that out of the plants that increased to a length of 1, 1.5, 2, and 3 cm, 1.5 cm long plants were the best suited for chromosome determination (Figure 2). This showed that the plant material assayed in this study to determine the chromosome numbers *Papaver* species was most active at this stage of growth showing active mitotic division for easy chromosome count. The roots tips were hydrolyzed by incubation in 1 N HCl at 60°C for 8 min followed by rinsing in distilled water for 2-3 min. to catch metaphase. This time (8 min) in hydrolyzing procedure is necessary for elimination of membrane cytoplasm. Hydrolyzed specimens were best stained with Feulgen for 30 minutes followed by washing with bi-distilled water before making preparates. Acetic acid was used to dissolve the fatty acid and waxy secretions, facilitating the penetration of the fixative. The observed chromosomes number is in agreement with previous studies showing the chromosome number of 42 in *P. pseudo-orientale*. Once the method was optimized, it was used to identify the rest of 68 samples.

Therefore subsequent experiments used 1.5 cm long plants for determination of chromosomes and identification of species.

A total of 69 samples were investigated. Their sample codes, subregion/region from where they were collected, period of germination (days), Germination (%), and species that were identified are given in Table 1.

Martens and Reisch [7] observed that the influence of the time of sampling in the activity of cell mitosis is very important. It is thought using ice cold water method for chromosome determination may have positively affected the roots tips during pretreatment ice cold water that helped in easy determination of chromosome counts at the time of mitotic division of root cells. It also increased the quality of the chromosome studies.

**Figure 3:**
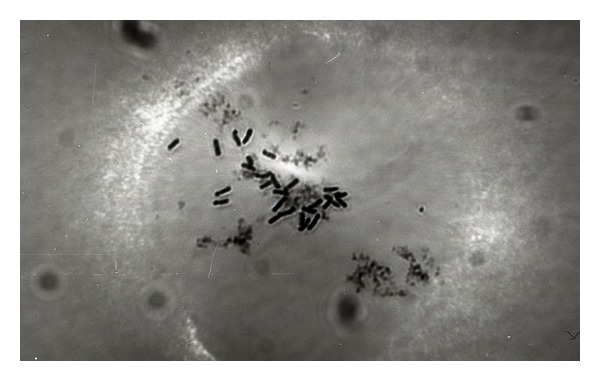
(10 × 40), 2*n* = 6*x* = 42 chromosomes belonging to sample 419 K of 446* P. pseudo orientale *collected from Caldıran, Gulhisar, and Van provinces, Turkey, pretreated with 0°C melting ice for 24 hours.

A review of the Table 1 shows that out of 69 samples, the seeds that belonged to seven samples (coded as 426, 435, 442, 478, 493, 495 and F1 (P.b × P.o) did not germinate and their chromosomes could not be counted (Table 1). Rest of the 62 samples except sample number 25 coded as 446 collected from Caldıran Gulhisar/VAN locations and sample number 67 coded as 7 collected from Iranian province of West Azerbaijan had chromosome number of 28 (Figure 3) and 14 and were identified as as *P. bracteatum* and *P. orientale*, respectively.

The *P. pseudo-orientale* seeds collected from different locations took 6–13 days to germinate. Whereas, the seeds of *P. bracteatum* took 8 days, *P. orientale* seeds took 10 days to germinate. Germination percentage of *P. pseudo-orientale* seeds was 2.38–90%, the seeds of *P. bracteatum* had seed germination percentage of 70%, and *P. orientale* had germination percentage of 25%.

Results of this chromosomal study have proven useful in plant taxonomy and phylogenetic analysis. In this sense, our results are helpful in identifying and separating *P. pseudo-orientale* species collected from Hakkari, Van, and Agri, and they noted no differences among them in terms of chromosome numbers. The results are in agreement with morphological features of the plants collected from these locations and previous studies by Goldblatt [1].

## Figures and Tables

**Table 1:** Sample code, sub region/region, number of days to germinate seeds, germination percentage, and the identified Papaver species from the samples collected from various regions of Turkey and Iran.

Number	Sample code	Sub-region/region	Period of germination (day)	Germination (%)	Species
1	414	Kayak T./Agrı	8	30	*P. Pseudo-orientale *
2	415	Kayak T./Agrı	8	30	*P. Pseudo-orientale *
3	416	Kayak T./Agrı	8	10	*P. Pseudo-orientale *
4	417	Kayak T./Agrı	7	90	*P. Pseudo-orientale *
5	419 K	Igdır yolu/Agrı	7	90	*P. Pseudo-orientale *
6	421	Hamur-Tutak/Agrı	6	60	*P. Pseudo-orientale *
7	423	Hamur-Tutak/Agrı	6	80	*P. Pseudo-orientale *
8	424	Hamur-Tutak/Agrı	10	60	*P. Pseudo-orientale *
9	425	Hamur-Tutak/Agrı	10	2.38	*P. Pseudo-orientale *
10	426	Hamur-Tutak/Agrı	—	0	Nil-
11	427	Hamur-Tutak/Agrı	8	40	*P. Pseudo-orientale *
12	428	Hamur-Tutak/Agrı	8	30	*P. Pseudo-orientale *
13	429	Hamur-Tutak/Agrı	11	10	*P. Pseudo-orientale *
14	430	Hamur-Tutak/Agrı	11	10	*P. Pseudo-orientale *
15	431	Hamur-Tutak/Agrı	11	10	*P. Pseudo-orientale *
16	432	Hamur-Tutak/Agrı	8	15	*P. Pseudo-orientale *
17	434	Hamur-Tutak/Agrı	7	70	*P. Pseudo-orientale *
18	435	Hamur-Tutak/Agrı	—	0	Nil-
19	437	Hamur-Tutak/Agrı	7	50	*P. Pseudo-orientale *
20	440	Hamur-Tutak/Agrı	9	70	*P. Pseudo-orientale *
21	441	Hamur-Tutak/Agrı	7	30	*P. Pseudo-orientale *
22	442	Hamur-Tutak/Agrı	—	0	Nil-
23	444 K	Safak cesmesi/Agrı	6	40	*P. Pseudo-orientale *
24	445	Caldıran So guksu/VAN	8	80	*P. Pseudo-orientale *
25	446	Caldıran Gulhisar/VAN	10	25	*P. Orientale *
26	448	Bahcesaray/VAN	8	60	*P. Pseudo-orientale *
27	449	Bahcesaray/VAN	8	70	*P. Pseudo-orientale *
28	450	Bahcesaray/VAN	6	90	*P. Pseudo-orientale *
29	451	Bahcesaray/VAN	7	2.38	*P. Pseudo-orientale *
30	452	Bahcesaray/VAN	7	90	*P. Pseudo-orientale *
31	453	Bahcesaray/VAN	8	90	*P. Pseudo-orientale *
32	454	Bahcesaray/VAN	8	90	*P. Pseudo-orientale *
33	455	Bahcesaray/VAN	8	90	*P. Pseudo-orientale *
34	456	Bahcesaray/VAN	6	80	*P. Pseudo-orientale *
35	457	Bahcesaray/VAN	7	90	*P. Pseudo-orientale *
36	458	Bahcesaray/VAN	7	90	*P. Pseudo-orientale *
37	459	Bahcesaray/VAN	8	90	*P. Pseudo-orientale *
38	462 K	Baskale Guzeldere/VAN	7	90	*P. Pseudo-orientale *
39	463	27 km to Baskale/VAN	7	80	*P. Pseudo-orientale *
40	465	Alan vadisi-Cesme yanı/HAKKARI	8	90	*P. Pseudo-orientale *
41	466	Alan vadisi-Cesme yanı/HAKKARI	8	70	*P. Pseudo-orientale *
42	468	Alan vadisi/HAKKARI	7	90	*P. Pseudo-orientale *
43	469	Alan vadisi/HAKKARI	8	70	*P. Pseudo-orıentale *
44	470 K	Alan vadisi/HAKKARI	7	70	*P. Pseudo-orientale *
45	472	Alan vadisi Karakol/HAKKARI	8	50	*P. Pseudo-orientale *
46	473	Alan vadisi Karakol/HAKKARI	8	60	*P. Pseudo-orientale *
47	475	Alan vadisi Karakol/HAKKARI	9	Very Low	*P. Pseudo-orientale *
48	476	Alan vadisi Karakol/HAKKARI	9	Very Low	*P. Pseudo-orientale *
49	477	Alan vadisi Karakol/HAKKARI	8	90	*P. Pseudo-orientale *
50	478	Alan vadisi Karakol/HAKKARI	—	0	Nil-
51	479	Alan vadisi Karakol/HAKKARI	10	90	*P. Pseudo-orientale *
52	480	Alan vadisi Karakol/HAKKARI	7	80	*P. Pseudo-orientale *
53	481 K	Alan vadisi Karakol/HAKKARI	7	90	*P. Pseudo-orientale *
54	486	Semdinli/HAKKARI	10	15	*P. Pseudo-orientale *
55	490	Yuksekova Semdinli/HAKKARI	8	50	*P. Pseudo-orientale *
56	491	Yuksekova Semdinli/HAKKARI	8	Very Low	*P. Pseudo-orientale *
57	492	Yuksekova Semdinli/HAKKARI	8	40	*P. Pseudo-orientale *
58	493	Yuksekova Semdinli/HAKKARI	—	0	Nil-
59	494	Yuksekova Semdinli/HAKKARI	7	70	*P. Pseudo-orientale *
60	495	Yuksekova Semdinli/HAKKARI	—	0	Nil-
61	496	Yuksekova Semdinli/HAKKARI	12	20	*P. Pseudo-orientale *
62	497	Yuksekova Semdinli/HAKKARI	13	10	*P. Pseudo-orientale *
63	500	Yuksekova Copluk/VAN	6	80	*P. Pseudo-orientale *
64	501	Yuksekova Copluk/VAN	8	70	*P. Pseudo-orientale *
65	503 K	Yuksekova kopru yanı/VAN	8	70	*P. Pseudo-orientale *
66	504 K	Dibekli Koprusu/VAN	8	90	*P. Pseudo-orientale *
67	7	Qushju Iranian province of West Azerbaijan, Iran	8	70	*P. bracteatum *
68	F1 (P.b × P.o)	Ankara University, Turkey	—	0	Nil
69	9	Qushju Iranian province of West Azerbaijan, Iran	9	90	*P. Pseudo-orientale *
